# Are there two forms of isometric muscle action? Results of the experimental study support a distinction between a holding and a pushing isometric muscle function

**DOI:** 10.1186/s13102-017-0075-z

**Published:** 2017-05-11

**Authors:** Laura V. Schaefer, Frank N. Bittmann

**Affiliations:** Section Regulative Physiology and Prevention, Department Sports and Health Sciences, University of Potsdam, Karl-Liebknecht-Str. 24-25, 14476 Potsdam, Germany

**Keywords:** Two forms of isometric muscle action, Holding isometric muscle action, Pushing isometric muscle action, Mechanomyography, Mechanotendography

## Abstract

**Background:**

In isometric muscle function, there are subjectively two different modes of performance: one can either hold isometrically – thus resist an impacting force – or push isometrically – therefore work against a stable resistance. The purpose of this study is to investigate whether or not two different isometric muscle actions – the holding vs. pushing one (HIMA vs PIMA) – can be distinguished by objective parameters.

**Methods:**

Ten subjects performed two different measuring modes at 80% of MVC realized by a special pneumatic system. During HIMA the subject had to resist the defined impacting force of the pneumatic system in an isometric position, whereby the force of the cylinder works in direction of elbow flexion against the subject. During PIMA the subject worked isometrically in direction of elbow extension against a stable position of the system. The signals of pressure, force, acceleration and mechanomyography/-tendography (MMG/MTG) of the elbow extensor (MMGtri/MTGtri) and the abdominal muscle (MMGobl) were recorded and evaluated concerning the duration of maintaining the force level (force endurance) and the characteristics of MMG-/MTG-signals. Statistical group differences comparing HIMA vs. PIMA were estimated using SPSS.

**Results:**

Significant differences between HIMA and PIMA were especially apparent regarding the force endurance: During HIMA the subjects showed a decisively shorter time of stable isometric position (19 ± 8 s) in comparison with PIMA (41 ± 24 s; *p* = .005). In addition, during PIMA the longest isometric plateau amounted to 59.4% of the overall duration time of isometric measuring, during HIMA it lasted 31.6% (*p* = .000). The frequency of MMG/MTG did not show significant differences. The power in the frequency ranges of 8–15 Hz and 10–29 Hz was significantly higher in the MTGtri performing HIMA compared to PIMA (but not for the MMGs). The amplitude of MMG/MTG did not show any significant difference considering the whole measurement. However, looking only at the last 10% of duration time (exhaustion), the MMGtri showed significantly higher amplitudes during PIMA.

**Conclusion:**

The results suggest that under holding isometric conditions muscles exhaust earlier. That means that there are probably two forms of isometric muscle action. We hypothesize two potential reasons for faster yielding during HIMA: (1) earlier metabolic fatigue of the muscle fibers and (2) the complexity of neural control strategies.

## Background

The human organism basically has two operation modes of muscle action: the static and the dynamic mode [1]. The dynamic working method can be categorized into concentric and eccentric muscle action. The static mode corresponds to the isometric muscle action [1–3]. Within concentric muscle action, we are able to overcome a resistance. In contrast, the eccentric muscle action enables us to decelerate an object. While isometric muscle action includes no gross joint motion, nevertheless, energy is consumed executing it. During performance of isometric muscle action in pilot studies, we have observed that two types of isometric muscle action can be executed. One can resist an object or one can push against it – although in both cases no motion is carried out.

The literature regarding different forms of isometric muscle action is very poor. Garner et al. [4] suggest that two forms of isometric action could exist based on different activity of electromyography (EMG) during concentric and eccentric muscle action. They hypothesize that the eccentrically loaded isometric muscle action (i.e., *holding isometric muscle action* (HIMA)) shows lower EMG-amplitude than the concentrically loaded one (i.e., *pushing isometric muscle action* (PIMA)). However, their readings of EMG-activity during muscle contractions up to 50% of the MVC of the soleus and gastrocnemius muscles showed no differences between HIMA and PIMA. Therefore, they reject the hypothesis and postulate that the modes of isometric muscle action are identical. Semmler et al. [5] also considered the possibility of two forms of isometric muscle action, but also found no statistical differences in EMG-activity.

Enoka and Rudroff et al. [6–8] used the term “force task” to describe a subject pulling or pushing actively against a stable structure (PIMA). A “position task” was defined as maintaining a constant position of the freely movable limb, while holding an inertial load (equivalent to the load during the “force task”), which represents HIMA. In summary, the two forms of isometric muscle action represents a position task (HIMA) that requires position control and a force task (PIMA) that requires the control of force.

Previous research suggests some objective differences between both hypothesized isometric actions. The most commonly found distinction is the “time to task failure” (i.e., the duration of maintaining either the position or the force task). Hunter et al. [9] performed experiments measuring fatigue of the elbow flexor muscles at 15% of the MVC in both of the isometric control tasks. The measurements showed a significantly longer duration time during the force task (PIMA) compared to the position task (HIMA). These findings were partially confirmed by Rudroff et al. [6, 7]. Similar results were only observed at lower intensities (20 and 30% MVC) [6, 7], when the arm was positioned horizontally. With higher force levels (45 and 60% of MVC) no differences between HIMA and PIMA were noted [7]. When the forearm was positioned vertically, no differences were found at any intensities [6].

Furthermore, the average EMG (AEMG) at exhaustion was greater during the force task (PIMA) compared to the position task (HIMA), despite a comparable rate of AEMG increase in both tasks. In contrast, the mean arterial pressure was higher at exhaustion during HIMA, while heart rate was not different [9].

Rudroff et al. [7] found significant differences in the spectral analysis of the power in the frequency band of 10 to 29 Hz. This bandwidth could be of particular interest, as the physiological frequencies of MMG-oscillations are found around 10 Hz. Thus, this could be another item to distinguish the hypothesized two forms of isometric muscle action.

Furthermore, Rudroff et al. [8] found a higher glucose uptake during HIMA compared to the PIMA in young men but not in older men.

Given that inconsistent findings regarding the two forms of isometric muscle action (HIMA vs. PIMA) we sought to examine this issue with a different methodological approach, which probably mimics real life situations better. Because there is freedom to vary position and force simultaneously in real life setting, our methodology aim to replicate that freedom.

It is hypothesized that two different forms of isometric muscle action (HIMA and PIMA) can be distinguished from each other based on the force endurance, the amplitudes of the mechanical myofascial oscillations (recorded by mechanomyography/-tendography; MMG/MTG) and the power spectral density (PSD). We hypothesize, due to previous research of Enoka et al. and pilot investigations at our department, that during PIMA the duration time is significantly longer than during HIMA. Furthermore, the mean amplitude of the whole duration time and the last 10% of duration time as well as the PSD of the MMG/MTG are hypothesized to differ significantly between HIMA and PIMA. Since it has been shown that EMG and MMG can synchronize and are therefore interdependent (e.g., [10, 11]), and furthermore Garner et al. [4] as well as Semmler et al. [5] did not found differences concerning the frequency of EMG comparing HIMA and PIMA, it is not expected to find differences in the mean frequency of the MMG/MTG either.

## Methods

### Participants

Ten healthy subjects (m = 5, f = 5) volunteered to participate in the study. The triceps brachii muscle on their dominant side was measured. All subjects were students of the University of Potsdam (studying sports therapy or sport for teaching profession), except for one participant, who was a high school student of age 18. Female subjects were aged averaged 24.4 years (±1.95), weighed 59.94 kg (±3.87) and were 169 cm tall (±1.58). They reached an averaged maximal voluntary isometric force (MVC) of 18.25 Nm (±1.96). The five male subjects were averagely 24 years old (±4.95), weighed 71.8 kg (±5.93) and were 182 cm tall (±3.81). They reached an averaged MVC of 31.166 Nm (±9.78). All subjects were right-handed. The study was conducted according to the declaration of Helsinki and local ethical permission was given. All subjects were informed in detail and gave their informed written consent to participate.

### Explanation of the terms holding and pushing isometric muscle action

The *Holding Isometric Muscle Action (HIMA)* designates the mode of isometry, during which the subject shall resist an applied external load in a static position. Accordingly, the subject has to apply as much force as is exerted from the exterior. Consequently, the position of holding isometric muscle action can only be maintained if the external load does not exceed the individual maximal force. If supramaximal forces are applied to the subject, the muscle action merges from isometric into eccentric muscle action. The *Pushing resp. Pulling Isometric Muscle Action (PIMA)* describes the mode of isometric muscle action, during which the subject pushes against or pulls at a resistance. However, the produced force does not overcome the given resistance. Since a constant muscle length is maintained during both modes, a lengthening (during HIMA) or shortening (during PIMA) of the muscle is prevented.

#### Realization of the holding and pushing isometric muscle action

The holding and the pushing isometric muscle actions were realized by means of the pneumatic system. Due to the compressibility of air the subject can *push* isometrically against the push rod to perform the PIMA. Thereby, the subject has to control the force and the position, since the push rod yields if the adjusted force is overcome by the subject, or extends, if the subject gives in. The pneumatic system is adjusted in a way that the pressure amounts to 80% of the pressure at the MVC of the subject when the push rod is pushed halfway into the cylinder. If the participant’s force is not increased or decreased, the pressure and the push rod stay stable. Thus, the subject’s task is to first push against the push rod until 80% of the MVC is reached and then to stay in this position.

However, the push rod can also push against the subject with a defined pressure. Again due to the compressibility of air, the subject is able to *hold* the push rod in a stable position, if the subject maintains just as much force as the pneumatic system applies externally. Thus, the maximal force of the subject must not be overcome. If the subject yields, the push rod moves out of the cylinder and if the subject pushes against the push rod, it is pressed into the cylinder and consequently in both situations the force, as well as the position, are no longer constant. The pneumatic system is adjusted individually in a way, that the subject reaches 80% of its MVC when the push rod is extended halfway. If the subject yields slightly, the pressure tends to decrease first, but is then readjusted by the system and increases automatically until 80% of the MVC are reached again. The instructed task for the subject is, to hold the out-coming push rod in the given position of 90° elbow flexion.

### Setting

**Fig. 1:**
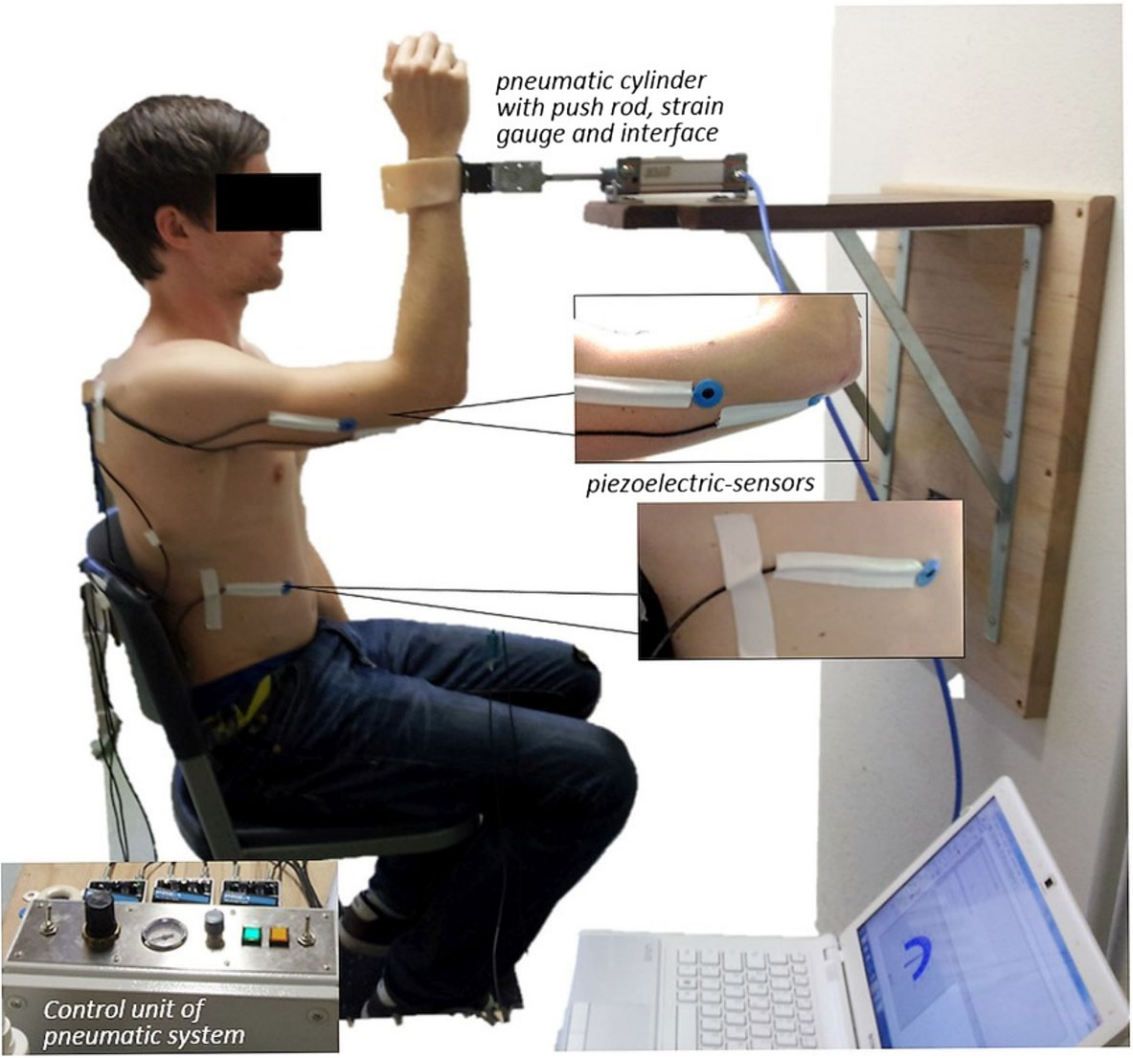
Setting for measuring HIMA and PIMA with the pneumatic system. The MMG/MTG of triceps brachii muscle and its tendon as well as the MMG of the abdominal external oblique muscle are recorded with piezo-sensors. The pressure is regulated with the control unit. All signals pass the A/D-converter and are recorded with DIAdem 10.2 on the measuring laptop

The setup was constructed especially for the measurements of the two isometric muscle actions (Fig. 1). The subject sits on a chair, while the angles between the subject’s leg and trunk, arm and trunk as well as the elbow angle measure 90°. The subject has contact to the pneumatic system via an interface, which is placed proximal to the ulnar styloid process. It was made sure, that the positioning was kept stable and reproducible during the whole measuring period. The interface consists of a shell made of a thermal deformable polymer material, which is commonly used in rehabilitation technology. Between the interface and push rod of the pneumatic cylinder a strain gauge is fixed (model: MLMZ 2000 N 36), in order to record the reaction force between the subject and the pneumatic system. Furthermore, an acceleration sensor (comp. Biovision) is attached to the strain gauge to detect the acceleration along the longitudinal acting force vector. In order to record the mechanic muscle oscillations, piezoelectric MMG-sensors (model: Shadow SH 4001) are applied painlessly with tape to the lateral head of the triceps brachii muscle, its tendon and the abdominal external oblique muscle. Due to the transversal muscle oscillation, the lateral positioning of the sensors on the skin above the muscle belly has an influence on the signal [12–15]. This fact, as well as the difficulty to standardize the contact pressure, has to be considered. That is one reason for the limited comparability of amplitudes between the subjects. Nevertheless, the amplitudes are comparable intrapersonally, because the sensors were not detached between the measurements of HIMA and PIMA. The MMG-signals are conducted across an amplifier (Nobels preamp booster pre-1) to an A/D-converter (comp. Biovision/National Instruments, 14-bit) and subsequently are recorded by the software NI DIAdem 10.2 (National Instruments on Sony Vaio: PCG- 61111 M, Windows 7). The sampling rate was 1000 Hz, therefore, the Shannon theorem was fulfilled. The subjects were able to control the produced pressure level via biofeedback at the beginning of the measurement on the laptop. Afterwards, the rater gave oral information, if the pressure level deviated.

#### Measuring procedure

In total 12 measurements were done: At first, two measurements with maximal intensity were performed against a stable resistance to determine the MVC of the subject. After this, each subject performed ten more measurements. The first six measurements were made for 15 s at 80% of the MVC. Thereby, three consecutive measurements for HIMA and PIMA were completed. The initial muscle action was randomized. Then the three measurement of each task were done in blocked order. The resting time between the trials was 60s. The endurance measurements followed. During this task, the subject had to maintain the position at 80% of the MVC for as long as possible performing either the HIMA or the PIMA. The initial muscle action was again randomized, followed by alternating HIMA and PIMA tasks. In this way, the influential factor of fatigue was minimized. The resting period was 120 s between endurance trials.

The abort criterion for all test trials was a deviation of the push rod of more than 3 cm from the middle position. Based on subjects’ varying arm lengths (range = 24.5…30.0 cm), 3 cm represents a change of 1.27°. This was considered to be irrelevant here. During the HIMA any change of the push rod in direction into the cylinder (pushing against it) was a failure criterion, because consequently the muscle action has turned from HIMA into PIMA. Failed trials were repeated.

### Data processing

The MMG-/MTG-, force-, pressure- and ACC-signals were recorded with the software DIAdem 10.2 (National Instruments) for all trials and were used for different considerations. The values of the strain gauge and of the pressure sensor during the given intensities were compared between the HIMA and PIMA to ensure the same force/pressure level. Torque was not calculated from the strain gauges’ force values as torque behaves proportionally to the force performing intraindividual comparisons.

**Fig. 2:**
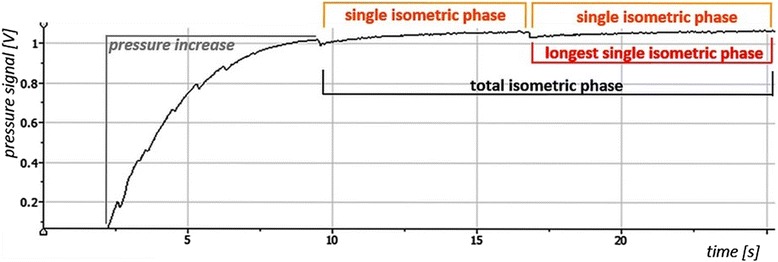
Exemplary curve of pressure signal during HIMA including the isometric parts. The different parts of the pressure signal were used inter alia for the cut of the other signals. The following parts can be differentiated: the pressure increase (*gray*), the total isometric phase (*black*), single isometric phases (*orange*) and the longest single isometric phase (*red*). The serrated deviations of the *line* show moments of jerky yielding

First of all, the isometric intervals of all raw signals were cut for further data analysis. They were excised based on the pressure signal (Fig. 2).

#### Isometric phases of the pressure signal for analyzing the force endurance

From the moment of reaching the given pressure level, the first deviation of the pressure signal (marked by a short visible pressure loss) was set at the starting point of the (first) isometric phase. The end of the first single isometric phase was appointed to the moment immediately before the second jerky yielding of pressure. These were so apparent in the pressure signal that a visual observation suffices. The next single isometric phase began immediately after the second deviation and this was continued (Fig. 2) until the subject falls below the given pressure level. Phases shorter than 1 s were not taken into account, because stable isometric condition were not built up. The sum of the duration time of all single isometric phases results in the total isometric phase. Furthermore, the longest single isometric phase of all signals was also used for specific considerations. Further preparations of data differ depending on the subsequent analysis.

#### Frequency of MMG-/MTG-signals

To evaluate the frequency of the MMG- and MTG-signals different methods were used: 1.) Power Spectral Density (Software DIAdem), 2.) Continuous Wavelet Transform (using Software Python), 3.) a Python script, which calculates the average of the time intervals between the maxima [16]. For 1.) and 2.) raw data were used. For 3.) the signal was filtered with a Butterworth filter (filtering degree 5, cut-off frequency: 20 Hz).

Furthermore, the power in the frequency range of 8 to 15 Hz, which is the commonly known physiological frequency of MMG, and the frequency band of 10 to 29 Hz, was investigated. The latter was considered by Hunter et al. [9] concerning the EMG power.

#### Amplitude of MMG-/MTG-signals

The data of the signals were filtered with a Butterworth filter (filtering degree 5, cut-off frequency: 20 Hz). Afterwards the arithmetic mean values of the amplitude of the total isometric phase and of the longest isometric phase were calculated using Excel (Microsoft Office). For another research question, just the last 10% of the duration time of the total isometric phase of the endurance measurements were included. This consideration of amplitude at exhaustion was done by Hunter et al. [9] concerning the AEMG. Regarding this analysis, two test persons had to be excluded, because their measurements at PIMA were stopped by the rater prior to complete exhaustion due to prolonged trial duration. For a further consideration, the coefficient of variation (CV) of the mean amplitude of the MMG-/MTG-signals between the three 15 s-measurements during the HIMA and the PIMA was calculated.

#### Group statistics

All group comparisons between HIMA and PIMA were done with SPSS Statistics (IBM). The significance level was set at α = 0.05 . Firstly, the data were tested by the Kolmogorov-Smirnov-Test regarding their normal distribution. If the normal distribution was fulfilled, the paired *t*-test was utilized. Otherwise, the comparison was done with the Wilcoxon-Test for paired samples.

Since we included several items in the testing procedure, the problem of multiple testing could be of relevance. This topic is controversially discussed in the literature (e.g., [17–19]). Rothman [20] claims that there are no adjustments needed for multiple comparisons. We basically support this opinion and agree with the conclusion of O’Brien [19]. Nevertheless, the problem of multiple comparisons seems to be considered particularly in clinical trials [21]. Since the present study is not a clinical trial, even proponents of adjustments for multiple comparisons might agree with our decision to set no adjustments here.

## Results

**Fig. 3:**
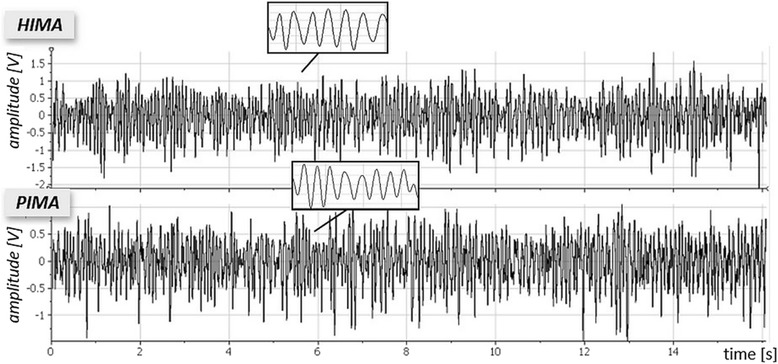
Exemplary signals of MMG of the triceps muscle. Displayed are the isometric phases of offset raw MMG signals of the triceps muscle during the holding isometric muscle action (HIMA; *above*) and during the pushing isometric muscle action (PIMA; *below*) at 80% of the MVC. The zoomed areas show a one-second sequence

Figure 3 displays exemplar raw signals of the MMG of the triceps brachii muscle during measurements at 80% of the MVC in the HIMA vs. PIMA.

### Force levels

The force level of the 15 s-measurements during HIMA and PIMA shows no significant difference. The mean force value of the holding isometric muscle action amounts to 99.31% of the PIMA. The mean force value of the strain gauge during HIMA was 55.19 N (±18.78) (men (m) = 67.18 N (±20.07), female (f) = 43.21 N (±5.65)). During the PIMA the arithmetic mean value of the strain gauge was 56.25 N (±20.94) (m = 69.24 N (±22.53), f = 43.25 N (±7.51)). The signals were distributed normally. The paired *t*-test was not significant (t(9) = 0.860; *p* = .412).

During the endurance measurements the force levels of HIMA resp. PIMA differ, although the pressure level does not. The mean force level of the *n* = 10 subjects during the HIMA endurance trials (55.14 N (±18.83)) amounts to 96.35% of the force level during the PIMA (57.23 N (±19.12); t(9) = 3,087; *p* = .013).

### Amplitude

The mean amplitudes of the MMG and MTG signals did not differ significantly between PIMA and HIMA (range of *p* = 0.069…0.765) regarding the total isometric measuring time, the longest isometric phase during the 15 s-measurements as well as during the endurance measurements.

Considering only the last 10% of the time duration of the endurance trials (exhaustion), the amplitude of the MMG of the triceps brachii muscle showed significantly higher amplitudes at exhaustion during PIMA comparing to HIMA (*n* = 8; W = −2.521, *p* = .012). The MMGobl and MTGtri did not show any substantial differences.

**Fig. 4:**
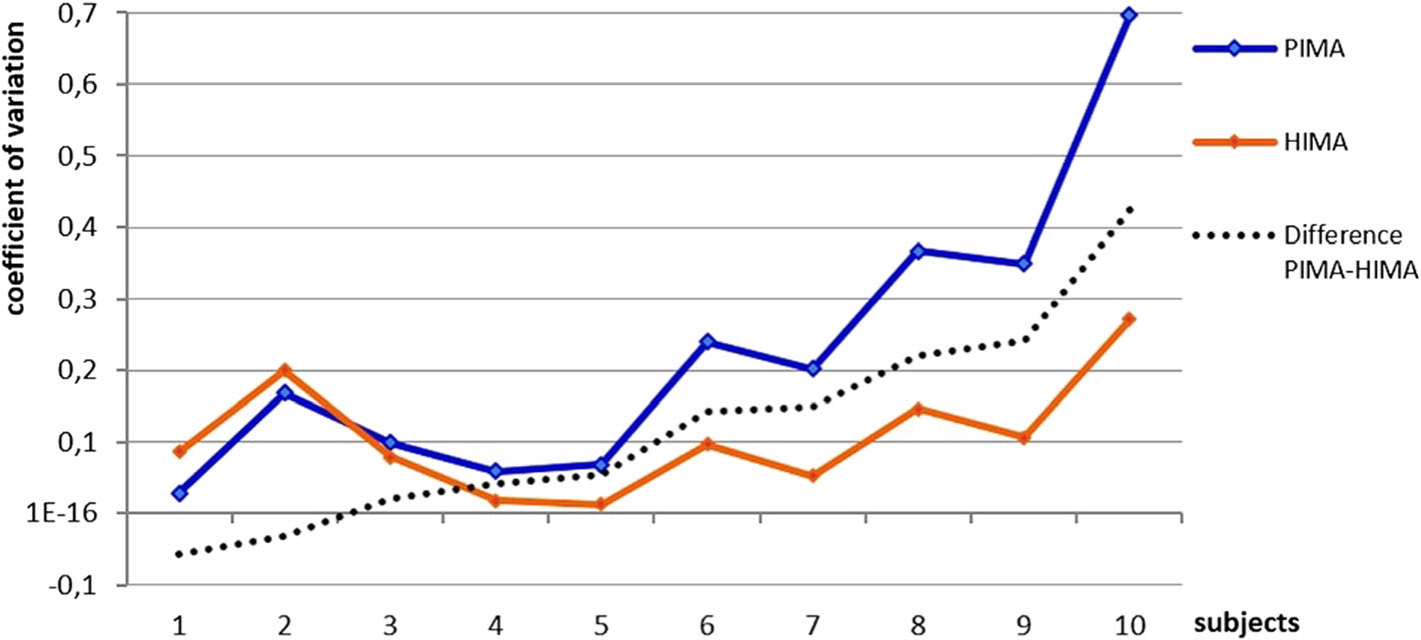
Coefficient of variation (CV) of the mean amplitude of MMGobl. Illustrated is the CV of the MMG amplitude of the abdominal external oblique muscle between the three 15 s-measurements of all *n* = 10 subjects during HIMA (*orange*) and PIMA (*blue*) sorted by the difference of the CV between PIMA and HIMA (*dashed line*)

During PIMA the CV of the amplitude of the MMGobl between the three 15 s-measurements (*n* = 10) was significantly higher comparing to HIMA (t(29) = 2523, *p* = .017) (Fig. 4). In eight of ten subjects the CV of mean amplitude of MMGobl (not in MMGtri or MTGtri) is higher during the PIMA.

### Frequency

**Fig. 5:**
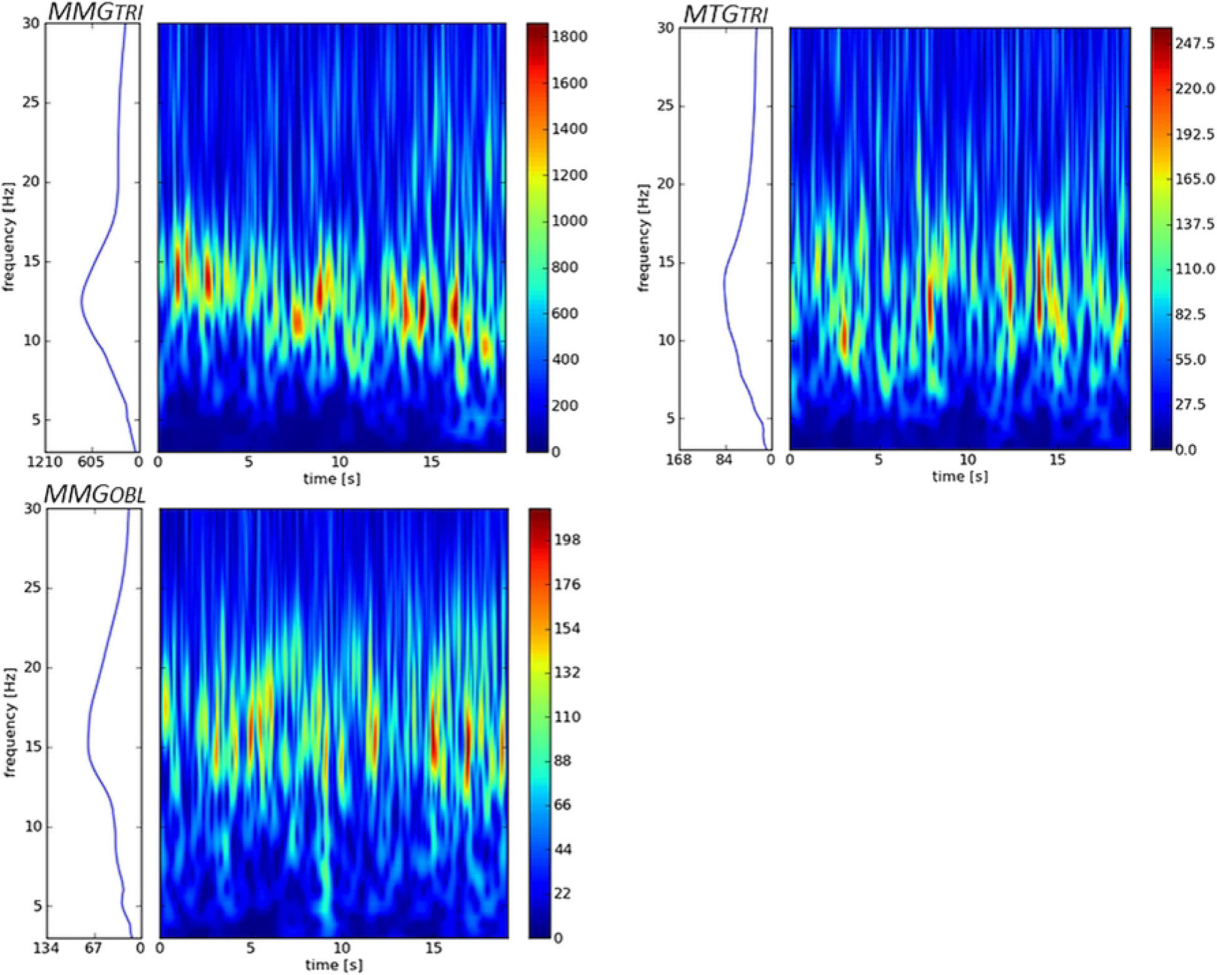
Schematic diagram of the Wavelet Spectra of the MMG/MTG-signals. The Wavelet spectra of the MMG resp. MTG of the triceps brachii muscle and its tendon (*top left/right*) as well as of the abdominal external oblique muscle (*bottom left*) during isometric muscle action at 80% of the MVC show the stochastically distributed oscillations in the low frequency area around approximately 15 Hz. The right scale displays the intensity of power. The *left side* of each figure displays the mean of the power over the time. This can be seen analogues to the Power Spectral Density (PSD)

Figure 5 shows the exemplary wavelet spectra of MMG and MTG-signals of one measurement. Since the HIMA and PIMA did not show any differences concerning these spectra, only the three spectra of one isometric mode are illustrated. The mean frequency shows no significant differences in the paired *t*-Test between the PIMA and the HIMA (*p* > .05).

### Power

Looking at the specific frequency ranges of 8 to 15 Hz and 10 to 29 Hz, respectively, the MTG-signal of the tendon of the triceps brachii muscle shows higher power values in HIMA than in PIMA. In the frequency range of 10 to 29 Hz the *t*-test for paired samples displays a significant difference (t(9) = −2.284, *p* = .048). This frequency range was chosen following Hunter et al. [9]. Looking at the common frequency range of MMG of 8 to 15 Hz, the significance becomes even clearer with *p* = .037 resulting from the Wilcoxon-Test for paired samples (power values were non-parametrically). The signals of the triceps brachii muscle as well as the abdominal external oblique muscle do not show any differences in either frequency ranges.

### Endurance time

**Fig. 6:**
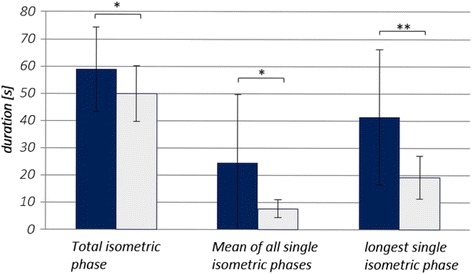
Items of the endurance measurements comparing the PIMA (*blue*) vs. HIMA (*gray*). The bars display the duration time [s] of different items of maintaining 80% of MVC comparing the pushing isometric muscle action (PIMA; *blue*) vs. the holding isometric muscle action (HIMA, *gray*). *p < .05, ** *p* < .01

**Fig. 7:**
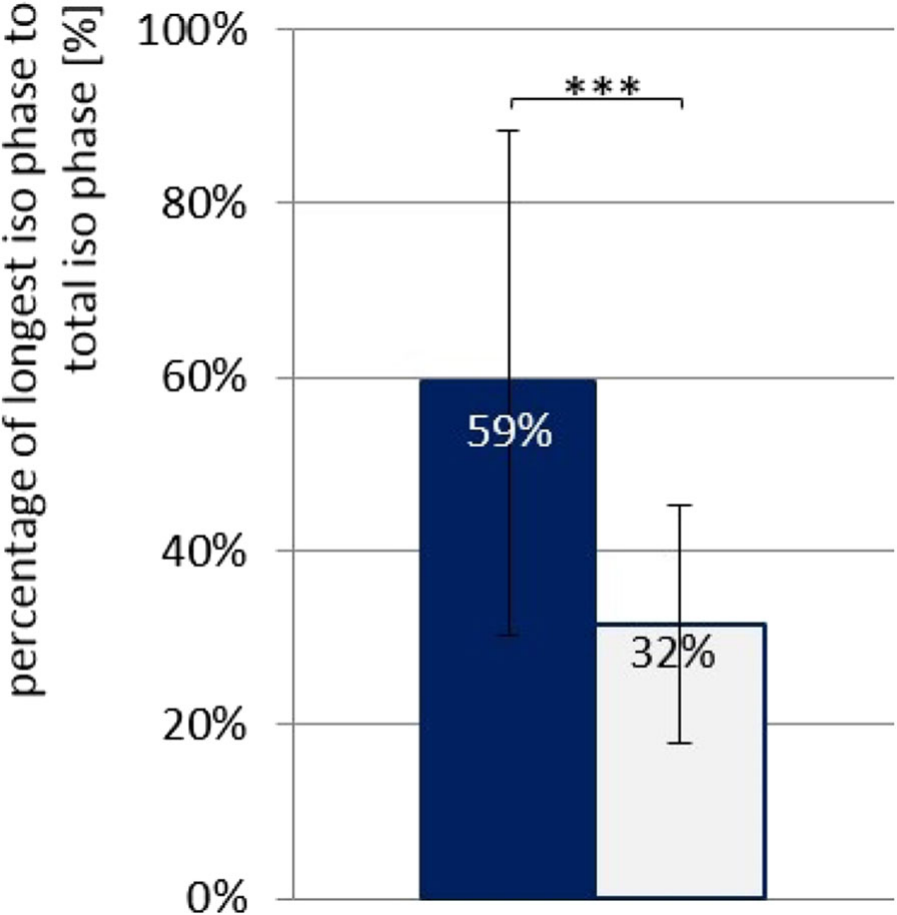
Relation between longest isometric phase and total isometric phase. Displayed are the arithmetic mean and the standard deviation comparing PIMA (*blue*) and HIMA (*gray*). ***p < .000

Using the pneumatic system, the single isometric phases of the duration measurements can be exactly identified, because any deviation of the push rod can be seen in the pressure signal (Fig. 2). Table 1 Therefore, the total isometric phase consists of several shorter single isometric phases. Table 1 shows the parameters’ raw values of the endurance trials. As can be seen in the last line and in Fig. 6, all items show significant differences between the PIMA and HIMA, respectively. During the pushing isometric muscle action, the subjects were able to stabilize a pure isometric position of the push rod (longest single isometric phase – without any deviation of the push rod) for approximately 41.4 s, which amounts to 59.4% of the total time duration of the isometric plateau (Fig. 7). During the holding isometric muscle action, the longest single isometric phase is 19.1 s, which relates to 31.6% of the total duration time. Thus, the pure isometric position in the PIMA could be maintained twice as long as in the HIMA.

**Table 1:** Overview of endurance parameters of the fatiguing measurements comparing PIMA vs. HIMA

	Total isometric phase [s]		Mean of all single iso. phases [s]		Longest single isometric phase [s]		Relation of longest single iso. phase to total iso. phase					
							PIMA		HIMA			
Vpn	PIMA	HIMA	PIMA	HIMA	PIMA	HIMA	M 1	M 2	M 1	M 2		
1	83.850	65.201	83.850	10.867	88.203	25.987	1.000	1.000	0.499	0.237		
2	61.890	59.962	11.770	7.270	28.176	14.017	0.489	0.292	0.095	0.309		
3	50.938	32.765	16.072	7.421	41.891	15.643	0.602	0.556	0.445	0.461		
4	38.742	49.067	15.494	7.284	25.257	16.222	0.616	0.613	0.251	0.361		
5	53.416	51.269	14.351	12.613	41.094	27.843	0.271	0.835	0.340	0.603		
6	60.284	48.820	6.694	3.394	19.274	10.189	0.199	0.339	0.148	0.229		
7	67.613	56.048	11.113	6.420	25.010	19.373	0.299	0.401	0.318	0.222		
8	37.853	41.237	19.040	6.206	44.280	18.976	1.000	0.822	0.379	0.329		
9	80.873	57.573	56.206	12.315	82.155	33.670	1.000	0.932	0.410	0.407	M1 & M2	
10	53.024	38.020	9.941	3.379	18.499	8.900	0.325	0.298	0.097	0.175	PIMA	HIMA
M	58.848	49.996	24.453	7.717	41.384	19.082	0.580	0.609	0.298	0.333	0.594	0.316
SD	15.526	10.250	25.128	3.282	24.896	7.929	0.320	0.273	0.145	0.131	0.290	0.136
CV	0.264	0.205	1.028	0.425	0.602	0.415	0.552	0.448	0.487	0.392	0.488	0.430
p	.029		.049		.005		.000					

Listed are the following items of each subject (Vpn; mean value of two measurements): the total isometric phase, the mean duration of all single isometric phases, the longest single isometric phase and the relation of the longest isometric phase to the total isometric phase. Furthermore the arithmetic mean (M), the standard deviation (SD) as well as the coefficient of variation (CV) and the results of the t-tests are displayed (α = .05). M1 = 1^st^ measuring, M2 = 2^nd^ measuring

## Discussion

### Content-related discussion

The present results provide findings, which partially support the hypothesis of two different forms of isometric muscle action. Overall, the authors assume the findings to be sufficient to distinct between the holding and the pushing isometric muscle action (HIMA vs. PIMA), respectively. Nevertheless, one also has to consider, that there are some items, which do not support the differentiation between such two forms.

The parameters, which show statistical differences between HIMA and PIMA, are the force endurance, the power in the frequency range of 8 to 15 Hz and 10 to 29 Hz of the MTG of the triceps tendon, the mean amplitude of the MMG of the triceps muscle at exhaustion and potentially the higher variability of the MMG of the abdominal external oblique. Therefore, the findings are generally consistent with them of Enoka and Rudroff et al. [6, 7, 9] as in the present study a longer force endurance appeared during PIMA. However, in contrast to their findings, the present study shows a significant difference between the HIMA and the PIMA regarding the endurance time even under higher intensities (80% of MVC) and with a vertically positioned forearm. One has to consider primarily, that their measurements were made in a different setting (no use of pneumatics, different forearm position), which could explain the different outcomes.

The oscillations of the MMG/MTG show inconclusive results regarding the hypothesis. The results do not display a statistically relevant difference between both isometric muscle actions concerning their frequency. This was expected based on the findings of other investigators (e.g., [4, 5]), who did not find differences concerning the frequency of the EMG.

The main question for us to discuss is how the lower force endurances during the HIMA, compared to the PIMA, can be explained. This also includes the findings concerning the oscillation characteristics (frequency and amplitude), since they may be interdependent. In general, different physiological processes could be responsible for faster muscular fatiguing. Weineck [22] lists several reasons, for example a central fatigue or a fatigue caused by reduced hormone production. These two possibilities can be excluded due to the short measuring time. The measurement duration also speaks against a faster synaptic fatigability in the HIMA. Furthermore, studies have shown that the EMG-activity is lower during eccentric muscle action (e.g., [23–25]). Assuming that the HIMA is closer to the eccentric muscle action (see below), this would also speak against a synaptic fatiguing. Thus, two potential sources of the faster yielding at submaximal static force endurance remain:

1. Metabolic fatigue of the muscle fibers (e.g., oxygen supply, removal of lactate)
2. Complexity of the neural control strategies.

The subsequent discussion is based on the hypothetical idea, that during the holding isometric muscle action (HIMA) the neuronal control strategies and muscle physiological aspects could be similar to the lengthening mode – analog to the eccentric activity – but without performing a change in length or joint angle. Vice versa, the mechanisms of the pushing isometric muscle action (PIMA) could be similar to the shortening mode – comparable to the concentric activity – without making a change in length or joint angle. On the one hand, this hypothesis is based on the subjective feeling during the performance of either the HIMA or the PIMA. On the other hand, this assumption could also be supported by the findings of Hunter et al. [9], who found greater AEMG at exhaustion during PIMA compared to HIMA. It is well known that during concentric muscle action, the amplitude of EMG is greater than during the eccentric one. So perhaps this could be a link between the proposed isometric muscle actions and the common eccentric and concentric ones. Garner et al. [4] also proposed this hypothesis. Furthermore, the study of Grabiner et al. [26] showed a higher muscle activation (EMG) if the subject expects a concentric contraction compared to if it anticipates an eccentric one. These different a priori activations of the CNS might have also occurred in the current investigation. The subjects were instructed to either hold the external resistance without allowing the push rod to overcome them (i.e., they were expecting an external resistance, which could force the subject into eccentric action, if the impacting force was not resisted sufficiently). Or the subjects were instructed to push against the push rod, which included the task to contract the muscle as in concentric muscle action, but not more than to a specific level of force. There are different approaches, which could support the hypothesis that HIMA is closer to eccentric muscle action, while PIMA corresponds to the concentric one instead. The following discussion grounds on this hypothesis.

#### Metabolic fatigue of the muscle fibers as reason for lower force endurance during HIMA

For the first mentioned aspect as a potential reason for the lower force endurance during HIMA – the metabolic fatigue (1) – a local lack of metabolic exchange under the conditions of intramuscular compression during isometric action could be presumed. This consideration is speculative as the current investigation did not measure substrate transport.

According to several authors [28–30] an ischemia of muscles can occur at intensities as low as 5–30% of MVC because the muscle is compressing its own blood vessels. Jensen et al. [31] reported a reduction of oxygenation at 30–40 mmHg, which corresponds to a contraction level of 20% of MVC. With intensities of 80% of MVC it is most likely that the blood vessels are also compressed, resulting in a probable ischemia. In this case it is plausible that not only the oxygenation, but also the exchange of substrates might be limited or not possible at all. The inadequate oxygenation due to the muscles’ compression would rather implicate an anaerobic metabolism during the present muscle tasks. Thereby, inter alia, lactate would be produced and would increase during the isometric muscle action. According to Katz et al. [32] this can amount up to 15 times of the initial value of lactate. Under these tensed up conditions during static muscle action, one has to ask, how an exchange and transport of substrates can be enabled at all over an interval of 50–60s? And why does the duration of force maintenance differ between HIMA and PIMA, even though the generated force, and thus the compression of muscle fibers, should remain the same? One possible explanation on how the exchange and transport of substrates is maintained could be based on the neuromuscular oscillations.

In the present study, the amplitudes of the MMG of the triceps muscle show higher values at exhaustion during PIMA compared to HIMA. EMG and MMG are different, but causally linked, methods with similar behavior at exhaustion (i.e., amplitude increase at higher intensities [11, 33–38]). Thus, one could expect related results. The investigations of Rudroff et al., Hunter et al. and the present study all show higher values at exhaustion concerning the mean amplitude during PIMA compared to HIMA in the AEMG [6–9] and in the MMG of triceps muscle, respectively (present study). The amplitude of the MMG reflects the fine mechanic oscillations of the muscle fibers. We presume that they are possibly necessary to maintain any of the exchange and transport of substrates during isometric muscle action. Because under particular circumstances the transport of fluids can be supported by vibration [39, 40]. The muscle tissue may possibly use a similar mechanism during reduced flow of substrates due to compression. Therefore, we hypothesize that higher oscillations – equivalent to vibrations – in the MMG of the triceps brachii muscle during the PIMA would probably support the substrate flow between the cell and interstitium as well as the flow within these areas. Conversely, lactate would probably be removed slower, and therefore would accumulate more during HIMA. Even though the lactate production is lower during eccentric muscle action [41, 42], the ratio between production and removal could potentially be more detrimental during the HIMA. Thus, the muscular fatigue could begin sooner during the HIMA and consequently the subject could yield earlier. Further investigation including measurements of lactate or O2 remain.

In fact, the hypothesis of exchange and transport of substrate could be a fundamental physiological reason for the obligatory requirement of muscular oscillations during isometric action in general. This could be an area of future research.

In the present measurements, it was impressively apparent, that during HIMA the position could not be maintained stable by the subjects for as long as during PIMA. The holding position was interrupted earlier by sudden, quick, but short motions of the push rod (and thus, short-time lengthening of the muscle). But immediately after these abrupt releasing actions, the position could again be maintained at the same level of force, but with a slightly changed elbow angle. This was merely a subjective observation, but maybe the exchange and transport of substances could be expedited by the jerky yielding of muscle tension (compared to a very short intensive oscillation). In this way, the metabolic environment might be improved again for a short time.

In view of these considerations it has to be taken into account, that only the MMG of the triceps brachii muscle shows a higher mean amplitude at exhaustion. The other sensor locations do not. Since the abdominal external oblique muscle is probably only utilized to stabilize the trunk, the different tasks of HIMA and PIMA may not be reflected there.

The question remains, why the triceps tendon does not show the same output as its muscle? Even though both methods, the MMG and MTG, measure mechanic oscillations, the mechanisms behind these oscillations are different. The working muscle generates oscillations actively, whereas the tendon just resonates in a passive way during force transmission. From this point of view, it is conceivable that at higher intensities, the muscle (motor) vibrates more intensively. In contrast, a more tightened tendon (like a passive rope) would swing with lower amplitudes (and possibly higher frequencies).

Almost no basic knowledge exists about the MTG. Based on our investigation we know that the oscillations of the MTG and MMG show similar frequency ranges and are able to generate coherent behavior during isometric muscle action [43, 44]. The authors are not aware of any other studies about the MTG. Thus, the MTG as a method is not yet established in science. Since the same sensors are used for MMG and the tendon is a passive structure which directly is connected to the muscle, the oscillations of the muscle theoretically has to be transmitted to the tendon. This is what we saw in previous investigations [43, 44]. Anyhow, we suppose that the tendon of triceps muscle occupies a kind of superposition, since all three heads, and a large number of motor units of the triceps muscle come together and form the oscillations. This, in turn, could be an explanation for the different outcomes regarding the power of the MTG resp. MMG signals. The MTG of the triceps brachii muscle show significantly higher power in the frequency range of 8 to 15 Hz (as well as 10 to 29 Hz) during HIMA compared to PIMA. We remember that the mean amplitude of MMG of triceps brachii was higher during PIMA at exhaustion. Normally, higher amplitudes indicate higher power. In this case one may ask, how these findings can be brought together?

#### Complexity of the neural control strategies as a possible reason for lower force endurance during HIMA

Among others, Semmler et al. [5] have shown that the synchronization of motor units (EMG) is higher during eccentric muscle action than during the concentric one. Possibly – and still under the assumption that the HIMA is closer to the processes of eccentric muscle action – a higher synchronization would enable the muscle fibers to oscillate in a narrower frequency range than during the PIMA. This could be the reason for the higher power of the MTG of the triceps in the low frequency range of 8 to 15 Hz (and 10 to 29 Hz) during the HIMA. While the PIMA is potentially wider spread, including other frequencies, and therefore results in lower power in the specific frequency range of 8 to 15 Hz. Rudroff et al. [7] found contrary results concerning the EMG. Their findings show that the power during the force task (similar to PIMA) was higher in 10 to 29 Hz than during the position task and not vice versa as found here.

Fang et al. [25] investigated the movement-related cortical potential (MRCP) via EEG comparing eccentric and concentric muscle action. They found that – although the EMG in their study showed lower amplitude during eccentric muscle action – the MRCP still resulted in greater values. The investigators postulated that eccentric muscle action is more difficult to perform compared to the concentric one, and therefore results in the higher brain activity. These findings are supported by the review of Enoka [27], who summarized, that the eccentric muscle action involves more complex control strategies of the nervous system than the concentric one. This was underpinned by Duchateau & Baudry [45], who added, that the unique mechanisms of eccentric muscle action are still unknown.

Bringing all of this together, the first paradoxically appearing result of significantly higher power in small frequency ranges during HIMA in the MTG, and conversely the greater amplitude at exhaustion during PIMA in the MMG of the triceps muscle could rely on the assumption, that eccentric muscle action is based on more complex neural control strategies. Thus, the present investigation indicates that HIMA could be more difficult to perform compared to PIMA due to a more complex adjustment of the neuromuscular system. Indicators for this are the shorter endurance time and the higher power of the MTG oscillations in a small frequency range of 8 to 15 Hz as a hint for a greater synchronization of muscle activation during HIMA. In turn, this possibly influences the fatigue mechanism, but further research is needed to test this hypothesis.

### Methodological limitations

In summary, we have to note two main methodological limitations within this study. Firstly, the force level shows a significant difference between HIMA and PIMA during the endurance measurements. We suppose that the reason for this lies in the stick and slip effect of the push rod. Since the push rod is not frictionless, jerky movements can occur during the slip out of the cylinder. Throughout the measurements, we controlled the force level of the subjects using the pressure signal, not the strain gauge’s signal. The reason for this was, that we had to use the pressure values to adjust the force of the pneumatic system individually for each subject to realize both measuring modes. While the pressure values do not show a statistical distinction between the HIMA and the PIMA, the values of the strain gauge do solely during the endurance measurements. Thereby, the stick and slip effect might appear to play a greater role when the piston is driving out of the push rod, which occurred more frequently during the endurance measurements compared to the 15 s trials. Therefore, prior to each yielding of the subject, the force level had to decrease. While the strain gauge captures this decline immediately, the push rod does not drive out instantly because of the friction. This may explain the different PIMA and HIMA pressure and force value behaviors. Anyway, the force needed to be maintained was lower during the HIMA, which should have made the task easier for subjects. As a result, this limitation may further support the results. A second limitation probably lies in identifying the maximal voluntary isometric contraction (MVC). As the setting shows, the subjects were fixed neither at their backs nor at their feet (Fig. 1). A higher MVC could possibly be generated with a different kind of setting, for instance by using a counterfort.

## Conclusion

The results seem to be sufficient for the authors to partially support the hypothesis of a differentiation of the isometric muscle action into two forms: The holding vs. pushing isometric muscle action (HIMA vs. PIMA). The results suggest that under holding isometric conditions muscles exhaust earlier. Further investigations have to be done on these findings. If the results will be supported, it has to be discussed how these postulated – but in motor science still not established – two different forms of muscle action can be included in the current theoretical construct of muscle activity. Currently, measurements concerning HIMA and PIMA with maximal intensities are in progress at our department. Furthermore, to obtain more detailed information concerning the assumption that HIMA is closer to eccentric muscle action, we have conducted first pilot measurements using EEG and MMG during HIMA and PIMA. Perhaps the hypothesis, that the amplitude in the EEG is higher during HIMA – analogues to eccentric – can be supported by this investigation. The evaluation remains ongoing. All this will probably deliver further hints for the control mechanisms of these specific muscle actions and the integration into the construct of operating modes of musculature.

The supposed differentiation of the two forms could probably bring benefits for diagnostics, prevention, and rehabilitation in the future. Since the eccentric muscle action implies specific control strategies of the nervous system, the supposed HIMA might require them as well. It is known, that during eccentric muscle action the vulnerability of the musculoskeletal system is at its highest [46–48]. Since the eccentric muscle action is based on a more complex neural control strategy, this also emphasizes the importance of a functionally intact neuromuscular system, while performing eccentric muscle action. It has to be investigated, whether or not HIMA is more sensitive for disorders of the neuromuscular system compared to PIMA, because of the higher vulnerability of eccentric muscle action. If the differences between the holding and the pushing isometric mode could be underpinned by further investigations, this differentiation would possibly lead to a more specific clinical muscle testing, e.g., used for neurological diagnosis.

In this regard, the authors also see potential in measuring two interacting neuromuscular systems to compare the two isometric muscle actions with each other [44]. This requires an even higher sensomotoric control of the neuromuscular system. Thus, it could deliver additional information about the control strategies during the two isometric muscle actions. The interaction, inter alia, will depend on the ability to synchronize the mechanical oscillations of both neuromuscular systems [44]. Results of these measurements of interacting partners concerning both isometric muscle actions will be presented soon.

## Abbreviations

ACC: Acceleration sensor
CV: Coefficient of variation
f: Female
HIMA: Holding isometric muscle action
m: Men
MMG: Mechanomyography
MMGobl: Mechanomyography of the abdominal external oblique muscle
MMGtri: Mechanomyography of triceps brachii muscle
MTG: Mechanotendography
MTGtri: Mechanotendography of the tendon of the triceps brachii muscle
MVC: Maximal voluntary contraction
PIMA: Pushing isometric muscle action
SD: Standard deviation
